# Multifunctionality is affected by interactions between green roof plant species, substrate depth, and substrate type

**DOI:** 10.1002/ece3.2691

**Published:** 2017-03-11

**Authors:** Yann Dusza, Sébastien Barot, Yvan Kraepiel, Jean‐Christophe Lata, Luc Abbadie, Xavier Raynaud

**Affiliations:** ^1^ Sorbonne UniversitésUPMC Univ. Paris 06IRD, CNRS, INRA, UPECUniv. Paris DiderotInstitute of Ecology and Environmental Sciences‐ParisiEESParisParisFrance; ^2^ Department of Geoecology and GeochemistryInstitute of Natural ResourcesTomsk Polytechnic UniversityTomskRussia

**Keywords:** ecosystem services, evapotranspiration, nitrogen and carbon cycles, soil–plant interactions, trade‐offs, urban ecology, water retention

## Abstract

Green roofs provide ecosystem services through evapotranspiration and nutrient cycling that depend, among others, on plant species, substrate type, and substrate depth. However, no study has assessed thoroughly how interactions between these factors alter ecosystem functions and multifunctionality of green roofs. We simulated some green roof conditions in a pot experiment. We planted 20 plant species from 10 genera and five families (Asteraceae, Caryophyllaceae, Crassulaceae, Fabaceae, and Poaceae) on two substrate types (natural vs. artificial) and two substrate depths (10 cm vs. 30 cm). As indicators of major ecosystem functions, we measured aboveground and belowground biomasses, foliar nitrogen and carbon content, foliar transpiration, substrate water retention, and dissolved organic carbon and nitrates in leachates. Interactions between substrate type and depth strongly affected ecosystem functions. Biomass production was increased in the artificial substrate and deeper substrates, as was water retention in most cases. In contrast, dissolved organic carbon leaching was higher in the artificial substrates. Except for the Fabaceae species, nitrate leaching was reduced in deep, natural soils. The highest transpiration rates were associated with natural soils. All functions were modulated by plant families or species. Plant effects differed according to the observed function and the type and depth of the substrate. Fabaceae species grown on natural soils had the most noticeable patterns, allowing high biomass production and high water retention but also high nitrate leaching from deep pots. No single combination of factors enhanced simultaneously all studied ecosystem functions, highlighting that soil–plant interactions induce trade‐offs between ecosystem functions. Substrate type and depth interactions are major drivers for green roof multifunctionality.

## 1 Introduction

While urban areas accounted for 54% of the world population in 2014 (United Nations 2014), there is growing evidence that urban ecosystems are crucial to tackle environmental issues in cities. Recent studies have shown that urban forests, rivers, wetlands, cultivated lands, grassland parks, and street greenery can provide multiple, scale‐dependant, ecosystem services. In contrast, green roofs have received less attention so far (Luederitz et al., 2015). As urban constructed ecosystems, they are able to provide multiple ecosystem services, such as thermal regulation of buildings, urban heat island mitigation, runoff water mitigation, water and air quality improvement, carbon storage, sound proofing, and biodiversity support (Oberndorfer et al., 2007).

Green roof design is constrained by load capacities of buildings, in particular when the installation of green roofs is decided after building construction. This need for lightness has led to the development of porous draining substrates based on light mineral components (Ondoño, Martínez‐Sánchez, & Moreno, 2016) such as pozzolan, a light and porous volcanic material. Green roof typologies are therefore based on substrate weight and depth. “Extensive” green roofs have thin substrate layers (usually <15 cm), need little maintenance but only allow for a low number of species. “Intensive” green roofs have thicker substrates (usually above 15 cm), can require more maintenance but allow for a larger diversity of plant species. Whatever the green roof type, the above‐described substrate characteristics, alongside with higher wind speed and sun exposure compared to ground (Cao, Tamura, & Yoshida, 2013), lead to frequent dry conditions for plants. Consequently, green roof vegetation is frequently based on drought‐resistant *Sedum* species that form the most widespread green roof systems when planted on shallow substrates (Vijayaraghavan, 2016).

Ecosystem functions in combination provide ecosystem services. For example, maintaining the long‐term fertility of green roofs, avoiding the release of polluted water, and storing carbon require closing nitrogen and carbon cycles. Similarly, evapotranspiration from green roofs participates in the mitigation of urban heat island effects. To date, studies of nutrients and water cycles in green roofs have mainly examined pre‐existing *Sedum*‐based extensive green roofs (Vijayaraghavan, 2016), but little is known on the relative influence of substrate composition, substrate depth, and plant species on the closing of carbon and nitrogen cycles, evapotranspiration, and water retention.

Substrate composition has been found to affect water quality, and it appears that more fertile substrates lead to higher carbon and nitrogen leaching rates (Beecham & Razzaghmanesh, 2015; Vijayaraghavan, Joshi, & Balasubramanian, 2012). More specifically, increasing nitrogen fertilization leads to more intense nitrate leaching (Clark & Zheng, 2014; Emilsson, Czemielberndtsson, Mattsson, & Rolf, 2007), while higher nitrogen contents induce higher plant growth (Clark & Zheng, 2014; Kanechi, Fujiwara, Shintani, Suzuki, & Uno, 2014; Rowe, Getter, & Durhman, 2012). Substrate composition also affects the amount of retained water. In natural soils, water retention is driven by the pore size distribution (Ding, Zhao, Feng, Peng, & Si, 2016). In contrast, green roof manufactured substrates (*e.g*., pozzolan, expanded clay) are designed to be highly porous to reduce their mass/volume ratio. Therefore, water retention not only depends on the interparticle pore size distribution, but also on intraparticle pore sizes (Graceson, Hare, Monaghan, & Hall, 2013).

Deeper substrates tend to lead to better plant growth (Nagase & Dunnett, 2010; Thuring, Berghage, & Beattie, 2010; VanWoert, Rowe, Andresen, Rugh, & Xiao, 2005). There is also evidence that increasing depth leads to higher water retention (Buccola & Spolek, 2010; Mentens, Raes, & Hermy, 2006). However, the role of depth concerning water quality is less consistent. Seidl, Gromaire, Saad, and De Gouvello (2013) observed that 16‐cm‐deep substrates led to higher nitrate and dissolved organic carbon (DOC) leaching compared to 6‐cm‐deep substrates. This study suggests that increasing substrate nitrogen and carbon content, through the increase in the total amount of substrate (mineral and organic matter), is likely to decrease water quality. In contrast, Retzlaff et al. (2008) and Razzaghmanesh, Beecham, and Salemi (2016) did not report any difference in nitrate concentrations of leachates from green roof systems when comparing, respectively, 5/10 cm and 10/30 cm depths. Soil depth could thus modulate the pollution of runoff water by other mechanisms. For instance, higher depths reduce water leaching, especially during low intensity rainfall events (Vijayaraghavan, 2016), leading potentially to more nitrogen and carbon holding. Effects of substrate on evapotranspiration are strongly related to water retention and substrate moisture content (Coutts, Daly, Beringer, & Tapper, 2013; Lazzarin, Castellotti, & Busato, 2005). Intriguingly, the direct impact of substrate depth and type on transpiration has never been assessed thoroughly, although it could influence plant transpiration efficiency through effects on water availability.

Little is known about the influence of plant species on green roof nutrient and water cycles. Lundholm, Tran, and Gebert (2015), using a plant functional trait approach, showed that higher plant biomass led to lower amounts of nitrate in soils, presumably because plants stored more nitrogen in their tissues. Because *Sedum* species are usually mat‐forming, shallow‐rooting plants, *Sedum*‐based green roofs should release high nitrogen concentrations. However, previous comparisons between species did not show clear patterns. For example, green roof trays planted with *Lolium perenne* (Poaceae) exhibited a higher nitrate release compared to green roof trays planted with *Sedum hispanicum* (Beck, Johnson, & Spolek, 2011). In contrast, another study reported much higher nitrate concentrations in leachate from *Sedum kamtschaticum* green roof modules, comparable to bare substrate leachates, than from two other succulent species (Aitkenhead‐Peterson, Dvorak, Volder, & Stanley, 2010). Nevertheless, all species increased the amount of released DOC when compared to a bare substrate. Plant species could also influence the water cycle. MacIvor and Lundholm (2011) found only weak differences of water retention between the bare substrate and the fifteen species (forbs, graminoïds, and creeping shrubs) they compared in monocultures, but a *Carex* species (Cyperaceae) and a *Danthonia* species (Poaceae) led to lower and higher levels of water retention, respectively. This suggests that plants might only play a marginal role on these processes compared to substrate, although Dunnett, Nagase, Booth, and Grime (2008), Dunnett, Nagase, and Hallam (2008) and Nagase and Dunnett (2012) observed that water retention was enhanced by species having higher biomass and height. When comparing evapotranspiration between bare substrates and plants, there is evidence that plant species may enhance total evapotranspiration (Ouldboukhitine, Belarbi, & Sailor, 2014). Blanusa et al. (2013) found that some large leaf herbaceous species can have higher stomatal conductances than *Sedum* species. However, to our knowledge, no author directly measured leaf transpiration in a green roof context.

Predicting the ecosystem services delivered by green roofs requires taking into account complex interactions between plant species, substrate type, and substrate depth and developing a multifunctional approach. Lundholm (2015) developed a multifunctional index for an extensive green roof and assessed that the global performance (including water retention, thermal regulation, nutrient uptake, and carbon sequestration) was enhanced by plant diversity. However, trade‐offs between functions, and by extension services, may occur. For instance, high nutrient content is expected to favor biomass productivity, and in turn total transpiration, but is also expected to increase nutrient leaching. In natural ecosystems, multiple functions are studied simultaneously to assess trade‐offs and synergies between ecosystem services and “disservices” (Butterfield & Suding, 2013), but to date, no study has focused on the influence of soil–plant interactions on trade‐offs between multiple ecosystem functions under green roof conditions.

This study aimed to evaluate the respective influence of soil–plant interactions on some major ecosystem functions determining important ecosystem services, such as urban heat island mitigation and limitation of runoff pollution. A second aim of this work was to determine whether these interactions lead to trade‐offs or synergies between ecosystem functions. This is an important step to provide general principles to design green roofs. We addressed these issues using a pot experiment under glasshouse conditions that allowed us to test 20 plant species, two substrate types, and two substrate depths in a full factorial design. A pot experiment cannot strictly reproduce the conditions experienced by full size green roofs, but it represents the only realistic way to implement 2 × 2 × 20 treatments with the desired level of replication. Despite their small size, microcosms are indeed useful in understanding larger scale ecological processes (Benton, Solan, Travis, & Sait, 2007) and pots with diameters as low as 100 or 110 mm have already been used to simulate green roof conditions (VanWoert, Rowe, Andresen, Rugh & Xiao, 2005; Durhman, Rowe & Rugh 2006; Wolf & Lundholm, 2008; Lu, Yuan, Yang, Chen, & Yang, 2015). We assessed the following functions: water retention, nitrogen and carbon storage in leaves, maximum leaf transpiration, aboveground and belowground biomass production, DOC, and nitrate leaching. These functions are related to C, N, and H_2_O cycling. They can thus be linked to ecosystem services such as runoff water quantity and quality, air quality, and urban heat island mitigation. Based on this experiment, our aims were to answer the following questions: (1) Do substrate type and depth affect ecosystem functions? (2) Do plant species modulate ecosystem functions? (3) Do soil–plant interactions lead to trade‐offs or synergies between ecosystem functions?

## 2 Materials and Methods

### 2.1 Soil material

Two substrate types were used. One was a commercial green roof substrate made of pozzolan and peat (i.D. Flore SP, Le Prieuré – Vegetal i.D., Moisy, France), thereafter named “artificial substrate.” The other was a natural sandy‐loam soil taken from a temperate grassland site (CEREEP‐Ecotron Ile‐de‐France, Saint Pierre‐lès‐Nemours, France), thereafter named “natural soil.” The natural soil was sieved (<5 mm) to remove roots, plant debris, and stones and then homogenized. Substrate characteristics are summarized in Table 1.

**Table 1:** Substrate characteristics (mean ± *SE*)

Soil characteristics	Natural soil	Artificial substrate
Type	Sandy‐loam	Pozzolan‐peat
Dry bulk density (kg/m^3^)	1.6 ± 0.01	1.1 ± 0.02
Saturated bulk density (kg/m^3^)	2.1 ± 0.03	1.5 ± 0.03
Water retention (% of dry soil)	33 ± 2.13	41 ± 2.99
C content (g/kg)	9.71 ± 0.26	51.14 ± 0.39
N content (g/kg)	0.74 ± 0.03	4.97 ± 0.04
pH	7.7 ± 0.09	7.4 ± 0.18

### 2.2 Plant material

We used 20 plant species that were known to have already been used on green roofs, belonging to 10 genera and five families (two genera per family, two species per genus). Species were all native to the Ile‐de‐France region (France) under subatlantic climate influence and were selected on their theoretical ability to perform well under dry conditions, based on their Ellenberg's indicators (Hill, Mountford, Roy, & Bunce, 1999). Species used were *Sedum album*,* Sedum acre*,* Hylotelephium telephium,* and *Hylotelephium maximum* for Crassulaceae; *Achillea millefolium*,* Achillea tomentosa*,* Centaurea jacea,* and *Centaurea scabiosa* for Asteraceae; *Cerastium alpinum*,* Cerastium tomentosum*,* Dianthus deltoides,* and *Dianthus carthusianorum* for Caryophyllaceae; *Festuca filiformis*,* Festuca glauca*,* Koeleria glauca,* and *Koeleria pyramidata* for Poaceae; and *Lotus alpinus*,* Lotus corniculatus*,* Trifolium fragiferum,* and *Trifolium repens* for Fabaceae. *Hylotelephium telephium*,* Cerastium alpinum,* and *Trifolium fragiferum* were removed from statistical analyses due to low germination rates.

### 2.3 Experimental design

PVC cylinders (125 mm diameter) having a height of 12 or 32 cm were used as pots. Bottoms were made of PVC plates, stuck with PVC glue, and drilled with five equidistant holes (1 cm diameter) to allow for drainage. Pots were filled with 10 or 30 cm of substrate, thereafter named “shallow” or “deep” treatments. Sixteen seeds were sown directly into pots on 17 July 2013 and thinned to only four plants per pot 2 weeks after germination. Five replicates were set up for each treatment combination making a total of 400 pots: 20 species × two soil depths × two soil types × five replicates. Pots were randomly placed in a glasshouse (CEREEP‐Ecotron Ile‐de‐France, Saint Pierre‐lès‐Nemours, France). To avoid shadowing effects from 30‐cm‐deep pots, 10‐cm‐deep pots were raised at the same level. Photoperiod was set at 15 hr a day with natural light and sodium lamps when natural light dropped under 200 W m^−2^ hr^−1^. Air temperature followed daily outside variations, but was maintained between 15 and 34°C. Plants were watered by hand directly onto the soil surface every 2 days with 200 ml of tap water.

### 2.4 Leaf transpiration

We used a portable infrared gas analyzer Li‐6400XT (Licor, Lincoln, Nebraska, USA) equipped with a CO_2_ mixer (6400‐01) and a chamber with an internal red/blue LED light source (LI‐6400‐02B) to measure maximum transpiration rates 5 months after plantation. For each treatment, measurements were performed on four replicates, on one leaf per pot left attached on the plant. The selected leaves exhibited the same light exposure and a similar size for each species. The whole measurement session lasted 4 weeks. To reduce time effects, a replicate of each treatment was measured each week. Time effects were tested with mixed‐effects models but were not significant. Transpiration was measured at 1200 μmol photons cm^−2^ s^−1^ under 400 ppm CO_2_, and leaf temperatures were kept at 24°C. Leaves were allowed to stabilize inside the measurement chamber for 2–4 min before each record. During the whole experiment, relative humidity was kept between 55% and 60%.

Leaves smaller than the 6 cm^2^ chamber surface were excised after measurement. For particularly narrow leaves (*e.g*., Poaceae), 5–10 leaves were placed inside the chamber. Transpiration being expressed as mmol H_2_O m^−2^ s^−1^, the leaves were photographed and their area determined using Image J software (Schneider, Rasband, & Eliceiri, 2012). To avoid high hydric stress of leaves, measurements were made only if soil moisture content in pots (ThetaProbe soil moisture sensor, Delta‐T devices, Cambridge, England) exceeded 0.1 cm^3^/cm^3^.

### 2.5 Runoff water quantity and quality

Water runoff quantity and quality were assessed through simulated rain events 6 months after plantation. Pots were slowly manually watered with 150 ml every 10 min, directly onto the substrate to avoid confusing effect of foliage interception, six times during 1 hr. They consequently received 900 ml in total, corresponding to an intense, although common for the Ile‐de‐France region, 18.3 mm/hr rain event. Soil moisture content was measured on the first 10 cm of substrate before watering (ThetaProbe soil moisture sensor, Delta‐T devices, Cambridge, England). Pots were placed on recipients filled with PEHD‐freezer bags. Once water stopped running‐off, bags were weighted. Water retention was expressed as the percentage of incoming water that did not run‐off. Runoff water was then homogenized and filtered (GF/F Whatman, Thermo Fisher Scientific, San Jose, California, USA). To measure DOC concentrations, 35 ml was collected and fixed with 35 μl orthophosphoric acid (85%). For nitrate (${\text{NO}}_{3}^{-}$) concentrations, samples were directly placed in −20°C freezer after filtration. DOC was measured with a total organic carbon analyzer (TOC‐VCSH; Shimadzu, Kyoto, Japan). Nitrates were measured by high‐performance liquid chromatography (ICS‐3000, Dionex, Sunnyvale, California, USA) equipped with an AS15 anion exchange column.

### 2.6 Biomass and leaf total carbon/nitrogen content

Seven months after plantation, plants were unpotted. Roots were recovered after water‐sieving. Roots and shoots were separately dried at 80°C during 2 days and weighted. Dry leaves of each pot were mixed and crushed for carbon and nitrogen analysis using a CHN elementary analyzer (Thermo Finnigan Flash EA1112, Thermo Fisher Scientific, San Jose, CA, USA). C/N ratios were calculated to evaluate how substrate type and substrate depth affect the availability of nitrogen and plant capacities to store carbon and nitrogen, which are related to important functions and services such as air and water quality.

### 2.7 Statistical analyses

Data analyses were performed using the R statistical software (version 3.2.2; R Core Team, 2015) with the significance level set at 5%. For biomasses, leaf carbon and nitrogen, water quality and retention, linear mixed‐effects models were fitted to all measures testing the effect of substrate type, substrate depth, and family (*nlme* package; Pinheiro et al. 2015). Genera and species were considered as nested random factors. Non‐normal data were transformed with log or cubic square roots when necessary. We used the r.squaredGLMM function (*MuMIn* package; Bartoń, 2015) to calculate marginal and conditional *R* ^2^ values and to obtain the part of variance explained by both fixed and random effects (Nakagawa & Schielzeth, 2013). Such models with families as fixed effects did not fit well for maximum transpiration (*R* ^2^m = 26%). We therefore used linear mixed‐effects models with substrate depth, substrate type, and plant species as fixed effects. Family and genera were considered as random effects.

Pairwise comparisons were calculated from these different models using the Tukey–Kramer method (*lsmeans* package; Lenth, 2015). For biomasses, leaf carbon, water retention, DOC, and nitrates in leachates, comparisons were performed between substrate treatments within each family, and between families within each substrate treatment. For transpiration, comparisons were calculated between plant species within substrate types, and between substrate types within each species.

Green roof multifunctionality and interactions between ecosystem functions were analyzed by principal component analysis (PCA) and between‐class analysis which can be seen as an exploratory generalization of the one‐way analysis of variance (*ade4* package; Dray & Dufour, 2007). Tests for between‐treatment differences were calculated from a Monte‐Carlo test (10,000 replicates) on between‐class analysis.

## 3 Results

### 3.1 Biomass production

**Figure 1:**
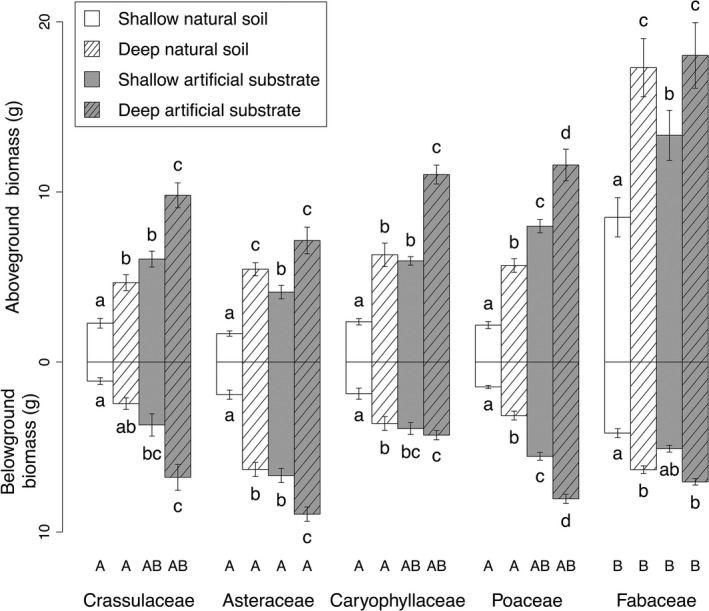
Average above‐ and belowground biomasses as a function of substrate depth and type (±*SE*). Biomasses from the different species were pooled for each family. Lowercase letters indicate differences (*p* < .05) between treatments within each family. Capital letters indicate differences (*p* < .05) in aboveground biomass between families within each type/depth treatment

Below, above and total biomasses were significantly affected by substrate depth and type, but only above and total biomasses showed significant differences between plant families (Table 2). Nonetheless, fixed factors explained 77% of variations for aboveground biomass, but only 36% for belowground biomass. For each family and each substrate type, plants grown on deep substrate exhibited higher aboveground biomasses (Figure 1) with more marked effects when growing on natural soil. On natural soil, biomasses from deep pots were two (Crassulaceae) to three times *(*Asteraceae) higher than from shallow pots, whereas biomasses from deep artificial pots were 1.4 (Fabaceae) to 1.9 times (Caryophyllaceae) higher than from shallow artificial pots. Depth effects were less obvious concerning root biomasses, without significant increase for Crassulaceae. Caryophyllaceae and Fabaceae showed an increase with depth only on natural soil, whereas only Asteraceae and Poaceae exhibited increases on both substrates.

**Table 2:** Squared‐R, degrees of freedom, F‐values, and significance for ANOVAs performed on fitted models. D stands for depth, S for substrate type, F for family, and Sp for species. Significance code for *p*‐values: .0001 “***”, .001 “**”, .01 “*”

Measures	Model		ANOVA degrees of freedom/*F*‐values/significance							
	*R* ^2^m	*R* ^2^c		Depth	Substrate	Family	D*S	D*F	S*F	D*S*F
Aboveground biomass	.77	.86	DF (num,den)	1,269	1,269	4,5	1,269	4,269	4,269	4,269
			*F*‐value	382.602	407.862	12.485	23.073	1.234	13.883	1.272
			Significance	***	***	**	**		***	
Belowground biomass	.36	.78	DF (num,den)	1,269	1,269	4,5	1,269	4,269	4,269	4,269
			*F*‐value	88.946	206.6120	0.604	2.163	1.156	10.549	1.687
			Significance	***	***				***	
Total biomass	.72	.83	DF (num,den)	1,269	1,269	4,5	1,269	4,269	4,269	4,269
			*F*‐value	291.311	410.051	6.352	17.245	1.333	13.061	1.581
			Significance	***	***	**	***		***	
Retention	.59	.60	DF (num,den)	1,279	1,279	4,5	1,279	4,279	4,279	4,279
			*F*‐value	140.293	21.048	23.481	3.981	3.982	7.747	10.660
			Significance	***	***	**	*	**	***	***
Dissolved organic carbon	.87	.88	DF (num,den)	1,280	1,280	4,5	1,280	4,280	4,280	4,280
			*F*‐value	74.867	1699.950	3.572	164.390	9.839	19.630	4.496
			Significance	***	***		***	***	***	***
Nitrates	.65	.69	DF (num,den)	1,253	1,253	4,5	1,280	4,253	4,253	4,253
			*F*‐value	29.008	79.571	20.448	81.823	4.943	10.646	11.830
			Significance	***	***	**	***	***	***	***
C/N ratio	.70	.76	DF (num,den)	1,274	1,274	4,5	1,274	4,274	4,274	4,274
			*F*‐value	22.332	0.797	57.010	3.330	3.534	0.376	0.662
			Significance	***		***		**		

	*R* ^2^m	*R* ^2^c		Depth	Substrate	Species	D*S	D*Sp	D*Sp	D*S*Sp
Transpiration	.58	.58	DF (num,den)	1,167	1,167	16,167	1,167	16,167	16,167	16,167
			*F*‐value	0.091	14.842	10.646	3.476	1.157	1.111	0.902
			Significance		***	***				

Artificial substrates led to higher aboveground biomasses, about two times for Caryophyllaceae*,* Crassulaceae, and Poaceae, than natural soil for each depth, except for Fabaceae and Asteraceae grown on deep pots. This substrate type effect was more pronounced for shallow depths with increases of 2.5 times (Caryophyllaceae*,* Crassulaceae*,* Asteraceae) to almost 3.5 times (Poaceae), although Fabaceae exhibited only an increase of about 1.5 times. For shallow depths, except Fabaceae, all families showed two times (Caryophyllaceae) to almost four times (Poaceae) higher belowground biomasses on artificial substrate compared to natural soil. These differences in belowground biomasses between both substrates were similar but weaker for deep pots, from 1.2 times (Caryophyllaceae) to 2.8 times higher (Crassulaceae).

Within each substrate treatment, few differences between families were detected. Aboveground biomasses of Fabaceae species were two to three times higher on natural soils than the other families, but were higher than only Asteraceae species on artificial substrates. No significant difference was found for belowground biomasses between families.

### 3.2 Water retention

**Figure 2:**
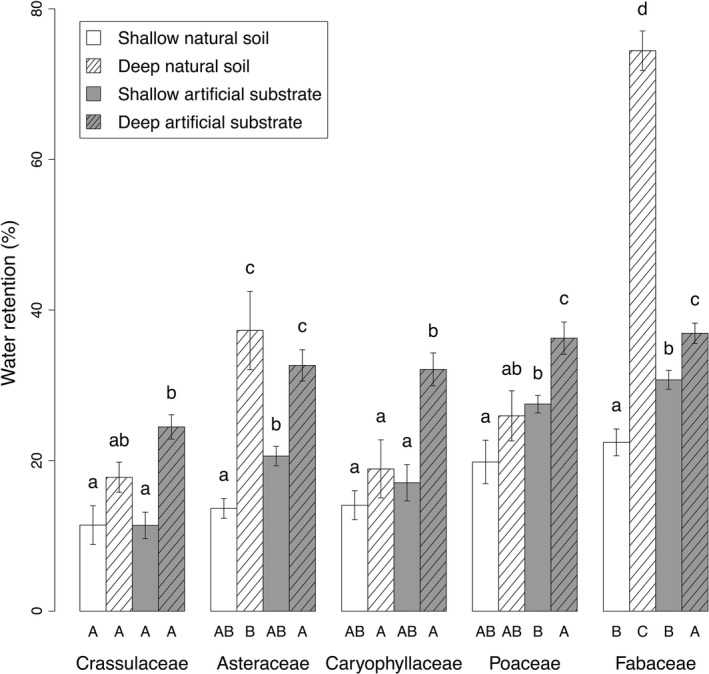
Average water retention as a function of substrate depth and type (±*SE*). Retention for the different species was pooled for each family. Lowercase letters indicate differences (*p* < .05) between treatments within each family. Capital letters indicate differences (*p* < .05) between families within each type/depth treatment

A significant negative relationship was found between substrate moisture content and water retention for each depth (see Figure S1 in Supporting Information). Water retention was significantly affected by substrate depth, substrate type, and plant family (Table 2). For each substrate type, increasing depth increased the amount of retained water. Differences were always significant for the artificial substrate, but highly variable, from 1.2 times (Fabaceae) to 2.2 times (Crassulaceae) higher for deep artificial substrate treatments than shallow artificial substrate treatments. For the natural soil, differences were only significant with Asteraceae and Fabaceae (Figure 2). Interestingly, Fabaceae pots exhibited the lowest increase in water retention with increasing depth for the artificial substrate, but the highest increase (more than three times) for the natural soil, even more than the Asteraceae pots.

Soil type effects were strongly dependent on plant family. For shallow depths, only Asteraceae, Poaceae, and Fabaceae exhibited higher significant water retention on artificial substrates, with an increase of about 1.5 times. For deep pots, when planted with Caryophyllaceae and Poaceae, the artificial substrate retained about 1.7 times and 1.4 times more water, respectively, than the deep natural soil. On the contrary, Fabaceae grown in deep natural soil retained twice as much water as the deep artificial substrate.

Considering comparisons between families within each substrate treatment, Fabaceae exhibited at least a doubling of water retention as compared to the other families in deep natural soil, but showed for both shallow treatments a retention that was only higher than the Crassulaceae. No significant difference was observed on the deep artificial substrate.

### 3.3 Dissolved organic carbon concentrations

**Figure 3:**
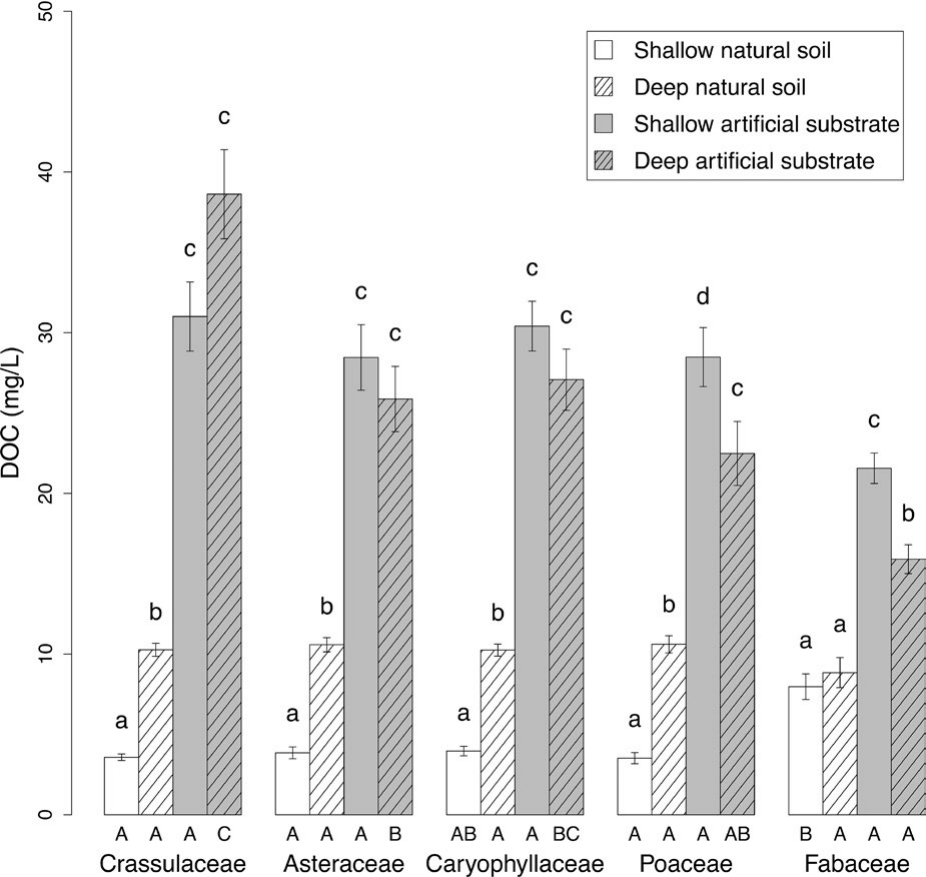
Average dissolved organic carbon (DOC) concentration in leachate as a function of substrate depth and type (±*SE*). DOC concentrations for the different species were pooled for each family. Lowercase letters indicate differences (*p* < .05) between treatments within each family. Capital letters indicate differences (*p* < .05) between families within each type/depth treatment

DOC concentrations of leachates were always significantly higher for artificial substrates than for natural soils (Table 2; Figure 3). On shallow substrates, the increases were of around seven to nine times for all families, except for Fabaceae (only two times). On deep substrates, differences were less pronounced: close to two times for Fabaceae and Poaceae, and up to three times for Asteraceae species (Figure 3).

Depth effects on DOC concentrations were substrate and family dependent. DOC concentrations were three times higher in deep than in shallow natural soils, except for Fabaceae. In contrast, DOC concentrations in the artificial substrate were about 1.3 times higher for shallow compared to deep pots for Fabaceae and Poaceae, while no significant difference between depths was observed for Crassulaceae, Asteraceae, and Caryophyllaceae.

Considering plant family effects within each substrate treatment, Fabaceae showed at least a doubling of DOC concentrations for shallow natural soils as compared to other families (although significance was not achieved with Caryophyllaceae). On the contrary, Fabaceae showed lower DOC concentrations in the deep artificial substrate, whereas the highest concentration was found for Crassulaceae. No difference between families was found for deep natural soil and shallow artificial substrate.

### 3.4 Nitrate concentrations

**Figure 4:**
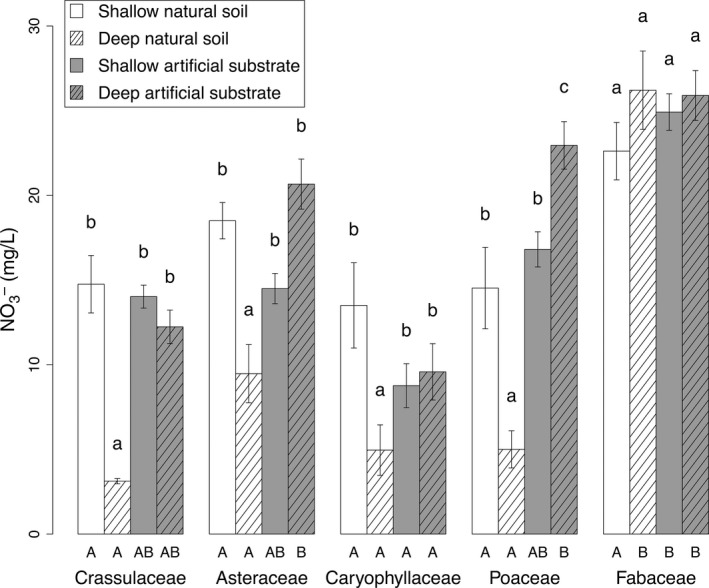
Average nitrate concentration in leachate as a function of substrate depth and type (±*SE*). Nitrate concentrations for the different species were pooled for each family. Lowercase letters indicate differences (*p* < .05) between treatments within each family. Capital letters indicate differences (*p* < .05) between families within each type/depth treatment

Nitrate concentrations in leachates were influenced by substrate depth, substrate type, and plant family (Table 2). All families except Fabaceae exhibited the lowest values on deep natural soil (Figure 4). No difference was found between other substrate treatments except for Poaceae. In this case, deep artificial substrates led to higher values than both shallow treatments. Fabaceae exhibited a very different pattern, with no difference between all the four treatments.

The analysis of plant family effects within substrate treatment showed no difference for nitrate on shallow natural soils, but Fabaceae exhibited two to eight times higher concentrations on deep natural soils than other families. For both depths of artificial substrates, Fabaceae showed higher nitrate concentrations than Caryophyllaceae.

### 3.5 Leaf transpiration

Transpiration was impacted by substrate type and plant species (Table 2). Transpiration rates were higher on natural soil for *Festuca filiformis*,* Koeleria glauca*,* Centaurea scabiosa,* and *Dianthus carthusianorum* (Figure S2).

Looking now at the plant species effect within each substrate treatment, transpiration rates differed between plant species grown on natural soil (*Dianthus deltoides*,* Sedum acre* < *Festuca filiformis*,* Festuca glauca*,* Koeleria glauca*,* Koeleria pyramidata*,* Achillea tomentosa*; Figure S2). Similarly, few differences between species were found on the artificial substrate (*Dianthus Carthusianorum*,* Dianthus deltoides*,* Sedum acre*,* Lotus alpinus*,* Achillea millefolium < Koeleria pyramidata,* and *Achillea tomentosa*).

### 3.6 Foliar carbon and nitrogen

Substrate depth and plant family significantly affected the leaf C/N ratio (Table 2). Depth effects were found only for artificial substrates and for three families (Crassulaceae, Caryophyllaceae, and Poaceae) with values about 1.2–1.3 times higher for shallow depths (Figure S3). The patterns for total nitrogen were similar to C/N ratio, but no difference was found for total foliar carbon except a slightly smaller value for Crassulaceae for shallow natural soil than for the other substrate treatments.

Considering plant family effects within each substrate treatment, Fabaceae always exhibited the lowest leaf C/N ratios (below 0.2, when other families were at least at 0.3; Figure S3). No difference was found between the four other families on both deep substrates. The shallow treatments induced similar patterns regardless of the type of substrate, Poaceae exhibiting 1.5 times higher ratios than Asteraceae and Caryophyllaceae.

Aboveground nitrogen stocks were estimated from species biomass values and respective foliar N content. They followed the same pattern as biomasses: Relatively less nitrogen was stocked in aboveground biomass for deeper substrates than for shallow ones (data not shown).

### 3.7 Interactions between functions

**Figure 5:**
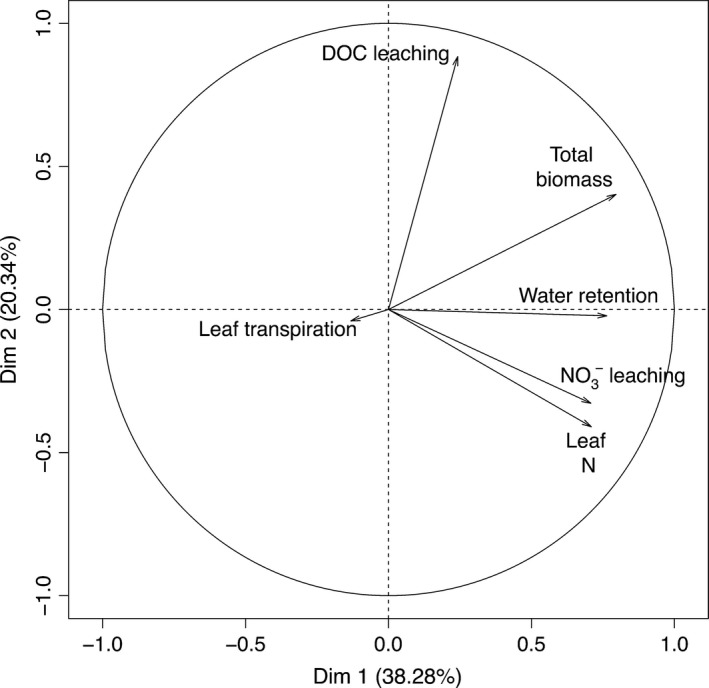
Correlation circle of the PCA computed on data of all ecosystem functions

**Figure 6:**
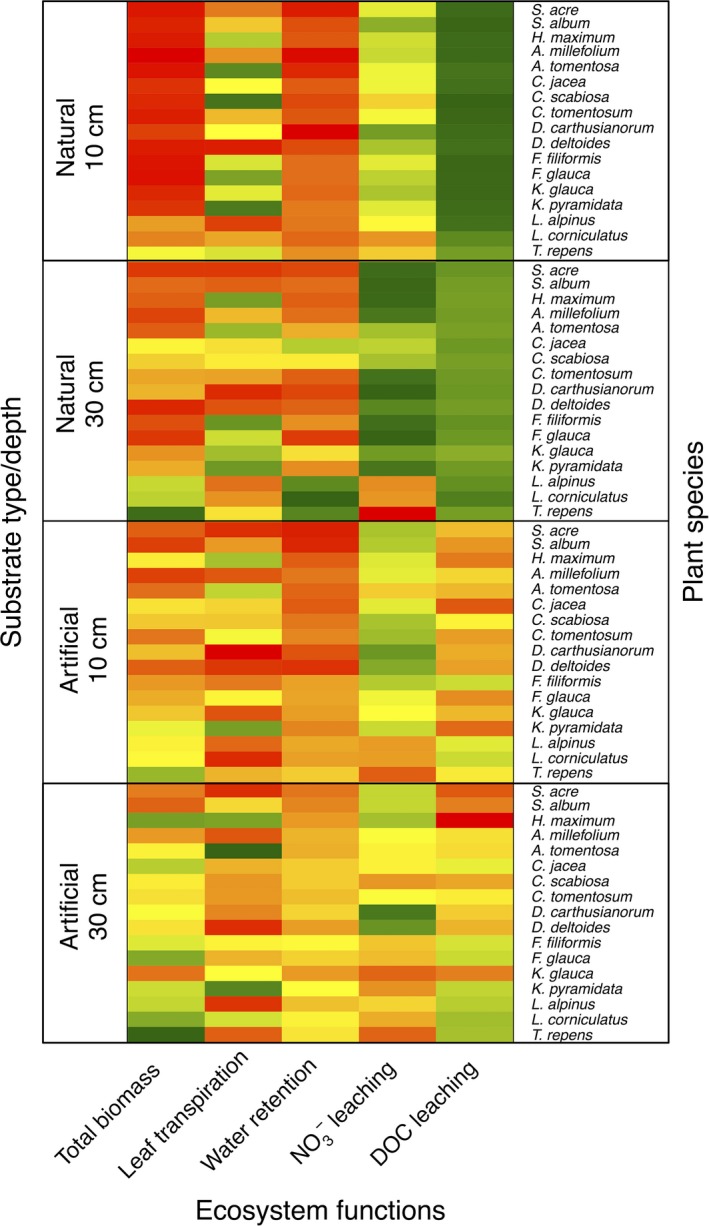
Heatmap of mean centered values of ecosystem functions for each treatment. The shading from red to green represents gradation from low to high relative performances. DOC and ${\text{NO}}_{3}^{-}$ leaching have been inversed compared to raw values, so high concentrations (*i.e*., low performance of cycle closeness) are shown in red

Principal component analysis indicated that nitrate leaching and leaf nitrogen were strongly correlated (Figure 5). DOC/${\text{NO}}_{3}^{-}$ concentrations in leachates and retention tended to increase with biomass. Between‐class analysis revealed that the four different substrate treatments (substrate type × substrate depth) were at different positions in the PCA space (*p*‐value <.0001, representing 24.3% of the total inertia). In order to synthesize visually our results, a heat map of the relative performance of each plant species/substrate type/substrate depth association for each studied function is shown in Figure 6.

## 4 Discussion

### 4.1 Green roof ecosystem functions are the results of substrate type/depth interactions

#### 4.1.1 Biomass production

Our results are consistent with several studies that showed a positive effect of increasing depth on plant biomass (Dunnett, Nagase, & Hallam, 2008; Durhman, Rowe, Building, Lansing, & Rugh, 2007; Lu et al., 2015; Thuring et al., 2010). Although we simulated dry environmental conditions, water was regularly provided. Therefore, the higher biomass on deep substrates is more likely due to a higher nutrient content rather than higher water content as increasing nitrogen availability has been shown to increase biomass (Clark & Zheng, 2014; Kanechi et al., 2014; Rowe et al., 2012). This is consistent with the fact that in our study, the deep artificial substrate pots that had the highest nitrogen content (the artificial substrate contained seven times as much nitrogen as the natural soil) led to the highest biomasses. Besides, increasing depth had stronger effects on natural soils and differences between both substrates were stronger with shallow substrates, suggesting that growth does not increase linearly with the amount of nutrients. These effects were less pronounced for belowground biomasses, suggesting that our species allocated more resources to aboveground structures when more nutrients were available. Interestingly, with the exception of Poaceae species, deep natural soil led to the same biomasses as shallow artificial substrate. This might be due to their fasciculate root system that, compared to taproots of other species, might have allowed a better exploration of substrate across its whole depth.

#### 4.1.2 Water retention

It has been shown that water retention increases with drier substrates (Bliss, Neufeld, & Ries, 2009; Stovin, Vesuviano, & Kasmin, 2012). This is consistent with our results, as we found a negative relationship between moisture content and retention for each depth. Multiplying substrate depth by three should have multiplied the number of pores by 3, although retention was three times higher only for Fabaceae and Asteraceae species on natural soil. For all other treatments, the retention capacity of a unit of substrate (total retention divided by the volume of substrate) was higher for shallow depths, suggesting that pore water content was higher before rain simulation for higher depths probably because shallow soils dried out faster. Overall, if positive effects of depth are usually found in the literature (Mentens et al., 2006), the size of this effect highly differs between authors and substrate depths. VanWoert, Rowe, Andresen, Rugh, Fernandez, et al. (2005) observed a slight increase of 3% of water retention when increasing depth from 2.5 to 4 cm for a 2% slope roof, while Buccola and Spolek (2010) found an increase of 36% from 5 to 14 cm. Besides, the size and intensity of rain events strongly influence water retention (Carter & Rasmussen, 2006), suggesting that the effects of substrate depth on water retention are likely to depend on the strength of the rain event.

#### 4.1.3 Dissolved organic carbon leaching mitigation

In all conditions, artificial substrate led to higher carbon concentrations in the leachates than natural soil, highlighting the strong initial differences in carbon content between the two substrates and suggesting weaker associations between mineral and organic matter in the artificial substrate. Studies focusing on DOC leaching from artificial substrates report high but variable concentrations. Berndtsson, Bengtsson, and Jinno (2009) found 12 mg/L DOC for an extensive green roof in Sweden (3 cm, crushed lava), but 40 mg/L for an intensive green roof in Japan (40 cm, perlite). Aitkenhead‐Peterson et al. (2010) and Beck et al. (2011) found concentrations of 40 and 75 mg/L of DOC, respectively, in leachates from 11‐cm‐deep green roofs. Our results for the artificial substrate are within the same range. Effects of substrate depth were less obvious. Whereas multiplying depth by three in natural soil led to a three times increase in DOC leachate concentrations, with the exception of Fabaceae species, increasing depth of the artificial substrate led either to smaller values or did not induce significant differences. A sufficient time of contact between sorbed DOC and water flow is necessary to allow for leaching (McTiernan, Jarvis, Scholefield, & Hayes, 2001). Water flow through the artificial substrate was rapid because of its high macroporosity (Figure S4). Consequently, each unit of substrate could have less contact with water in the deep substrate. On the contrary, the natural sandy‐loam texture substrate was likely to retain water longer because of smaller pores, allowing the deep treatment to leach proportionally as much DOC as the shallow substrate. Alternatively, shallow substrates were more likely to dry, thus producing more DOC because of higher rate of microbial lysis (Lundquist, Jackson, & Scow, 1999).

#### 4.1.4 Nitrate leaching mitigation

According to the literature, reported nitrate concentrations in leachates are highly variable and primarily depend upon the initial nitrogen quantity (Emilsson et al., 2007). Beecham and Razzaghmanesh (2015) compared different substrates and depths. A 30‐cm substrate based on scoria led to 1.75 mg/L of nitrates in leachates, while the same substrate enriched with organic matter led to 10 mg/L of nitrates. However, similar to our results for the artificial substrate and using the same substrate depths, they did not find depth effect. For other authors, increasing depth has been shown to either increase nitrate concentrations (Seidl et al., 2013) or to have no effect (Retzlaff et al., 2008). The very low nitrate concentration we observed in leachates from deep natural soils was an intriguing result. The relatively lower storage of nitrogen in aboveground biomass for higher depth substrates compared to shallow depth suggests that nutrients were no longer limiting factors in deep substrates, leading to less nitrate uptake by unit of substrate volume. Similarly, root density (biomass of root per unit of substrate volume) was higher for shallow than for deep artificial substrate, suggesting a possible higher mineral nutrition per unit of soil volume in shallow substrates. A higher initial nitrogen stock and a weaker relative nitrate uptake on deep soils should have allowed more nitrate leaching, but this was not the case.

We measured ammonia along with nitrates, but we could not analyze these data due to high numbers of samples under detection limit. Nonetheless, ammonia was only detectable in samples from deep natural soil, whose nitrate concentrations were the lowest, suggesting that nitrification occurred at reduced rate in this treatment. This could be due to a lower rate of water loss during the whole experiment, deeper layers experiencing less aerobic conditions thus limiting nitrification (Emilsson et al., 2007), while increasing denitrification rates.

#### 4.1.5 Foliar transpiration

In our experiment, effects of the substrate type were not due to the immediate water status of substrates, as no difference in soil moisture content was found. The higher leaf transpiration rate on natural soil might be linked to smaller biomasses and thus leaf area index, inducing a smaller total water consumption and more available water for each leaf (Albert et al., 2012). Alternatively, the drier conditions induced by the artificial, draining substrate might have led to drought adaptations strategies, such as reduced stomatal densities (Yoo et al., 2010).

### 4.2 Modulating green roof functions through plant families and species

Except for transpiration, we chose to group plant species and genera to analyze the effect of families. Hence, it was not possible to test the effects of species or genus. The lack of family effect concerning biomass was likely to be due to low homogeneity within families. For instance, although the Crassulaceae species showed many similarities (succulent, shallow‐rooting), *Hylotelephium* species formed high epigeous structures, whereas *Sedum* species were mat‐forming. Besides, the use of pots might have resulted in some edge effects, especially concerning belowground biomass. Some shallow‐rooting species such as Crassulaceae species may have been less impacted by such effects than deep‐rooting species such as Asteraceae species. Overall, we found that Fabaceae species have an interesting potential for green roofs. Fabaceae grown on natural soils showed higher aerial biomasses than other families, reflecting their ability to fix nitrogen as leguminous species on poor medium. On deep natural soils, Fabaceae led to two to three times higher water retention and nitrate leaching than other plants, suggesting a faster drying of substrate and high nitrification induced by these aerobic conditions; this family also led to higher DOC concentrations, maybe due to higher production of exudates. To date, green roof substrate microbial communities have not received much attention, although both their structure and functioning might be affected by green roof conditions (Ondoño, Bastida, & Moreno, 2014). We showed with the principal component analyses that higher biomasses were associated with more water retention. This is consistent with the findings of Nagase and Dunnett (2012) and Lundholm et al. (2015) who found that higher plants were associated with higher retention rates. In particular, under the deep natural soil conditions, Fabaceae species may have experienced a higher water requirement to sustain the higher biomass.

Few significant differences were observed between species for leaf transpiration. On natural soil, the lowest transpiration rate was found for two species having a low growth form (*Sedum acre*,* Dianthus deltoides*), as it had been observed in natural ecosystems (Körner, Scheel, & Bauer, 1979). Farrell, Szota, Williams, and Arndt (2013) estimated total transpiration by comparing the weights of bare pots and pots planted with various species. Although technically the loss of water due to plants cannot fully be attributed to transpiration, as plants may have opposing effects on evaporation and transpiration (Lundholm, MacIvor, MacDougall, & Ranalli, 2010), these authors showed that plant species had strong and varied effects on total water loss. In the future, combining such an approach with detailed foliar gaseous exchanges and under various substrate moisture contents would be useful to understand trade‐offs between evaporation and transpiration and how plant species affect the resulting ecosystem services (mainly thermal and water regulation). Finally, few differences were found between species concerning foliar and nitrogen content, except for Fabaceae species, whose high N content was due to its capacities to fix nitrogen.

### 4.3 Trade‐offs between ecosystem functions

Lundholm (2015) showed that various monocultures or mixture treatments were associated with different levels of functions (mainly substrate temperature reduction and water retention). This is consistent with our results summarized in Figure 6 that show that all functions cannot be maximized simultaneously, thus suggesting that trade‐offs are likely to occur between different ecosystem services on green roofs as a result of soil–plant interactions, as it is already documented for natural ecosystems (Butterfield & Suding, 2013). For instance, if urban heat island mitigation is an important aim, high transpiration species such as *Koeleria pyramidata* on deep substrates are to be preferred due to high biomass and high soil water retention capacities thus leading to higher evapotranspiration. If water retention during rainstorm events is to be promoted, then species with denser root systems such as Fabaceae species on deep natural soil are likely to be more efficient. On the contrary, limiting nitrate pollution implies using the same soil treatment, without Fabaceae species, while DOC pollution is to be mitigated with shallow natural soils.

Research under real green roof conditions is required to test the robustness of our findings on the impact of soil–plant interactions on trade‐offs between ecosystem services. For instance, biomass, foliar transpiration, vegetation cover, leaf area index, and water retention need to be jointly taken into account to predict evapotranspiration which, depending on environmental conditions, influences urban heat island mitigation. Our experiment was short term, but long‐term evolution of green roofs is also a key issue. For example, high nutrient leaching after the installation of a green roof might lead to impoverished substrates after a few years thus altering the provision of ecosystem services.

Finally, our substrates had various bulk densities and the heaviest (deep natural soil), which was often associated with the most efficient treatments, might not be applicable on many existing buildings. Although shallow depths can also induce high function levels, they might nonetheless lead to plant stress and reduced efficiency under prolonged drought on roofs (Van Mechelen, Dutoit, & Hermy, 2015), thus requiring some watering to maintain multifunctionality. This is particularly true for the artificial substrate that had the highest maximum retention, but dried faster (Figure S4).

Taken together, our results suggest that the provision of ecosystem services by green roofs may result from complex interactions between substrate type, substrate depth, and plant species and that these interactions likely lead to trade‐offs between services. Although green roofs have been initially installed on existing buildings, the development of environmental policies in cities will lead to the emergence of new green buildings. More multifunctional green roofs should emerge if multifunctionality is addressed during the conception stage, allowing a wider variety of substrate types, substrate depths, and plant species.

## Conflict of Interest

None declared.
