# Supplementary material for: Multifunctionality is affected by interactions between green roof plant species, substrate depth, and substrate type
Source: Ecol Evol. 2017 Mar 11;7(7):2357–69. doi: 10.1002/ece3.2691 (PMC5383477; doi:10.1002/ece3.2691)

**Figure S1. Relation between water retention and substrate moisture content measured on the first 10 cm before the watering experiment.** '***' stands for significance (p-value<0.0001). **a.** Relation for the shallow substrates. **b.** Relation for the deep substrates.

a.

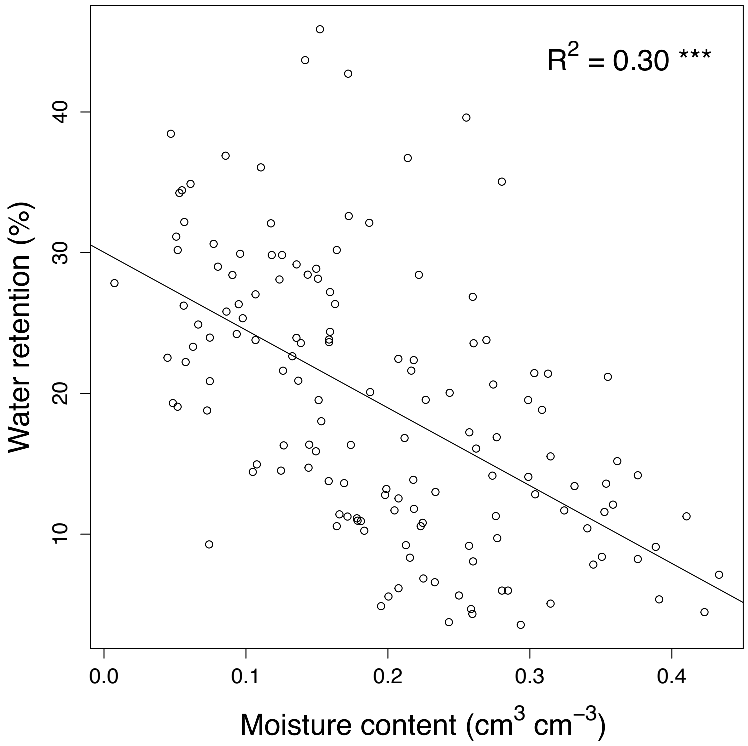


b.

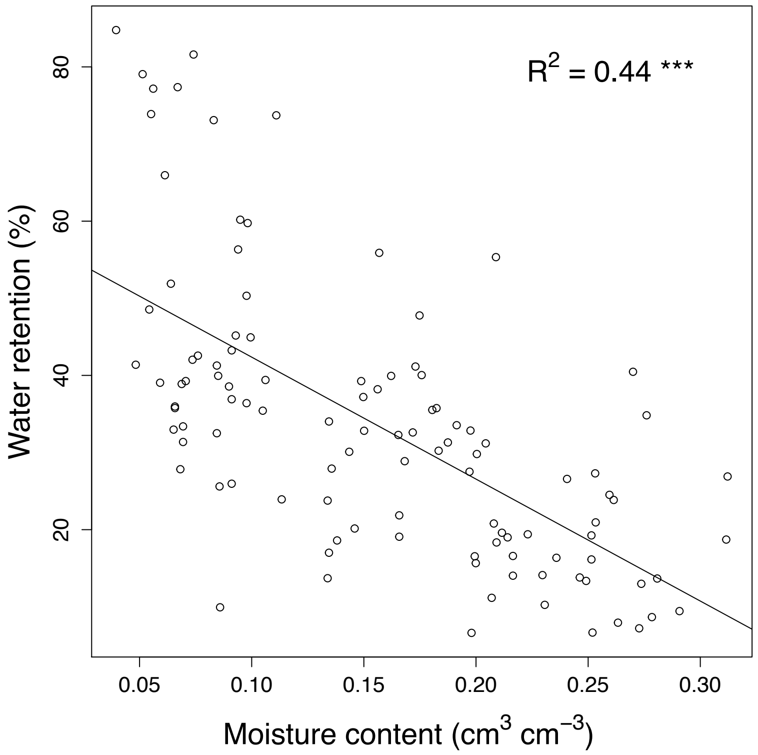

Supplement: Supplementary file 1 [file ECE3-7-2357-s001.pdf]
