# Supplementary material for: Multifunctionality is affected by interactions between green roof plant species, substrate depth, and substrate type
Source: Ecol Evol. 2017 Mar 11;7(7):2357–69. doi: 10.1002/ece3.2691 (PMC5383477; doi:10.1002/ece3.2691)

Figure S2. Average maximum leaf transpiration rates as a function of substrate type (±SE). Transpiration rates were pooled for each species. Lower cases letters indicate differences ($p<0.05$) between treatments within each species. Capital letters indicate differences ($p<0.05$) between species within each substrate type.

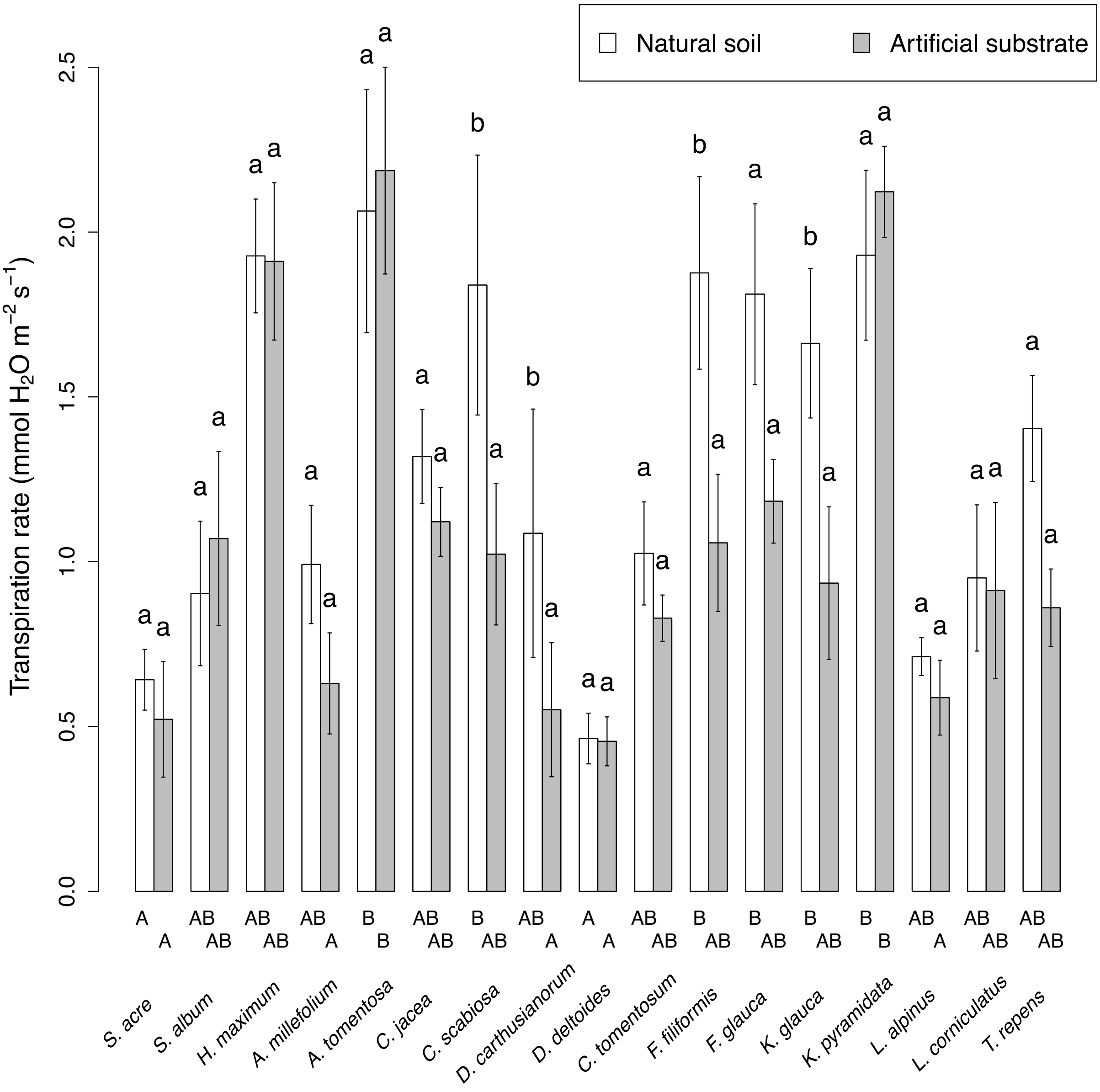

Supplement: Supplementary file 2 [file ECE3-7-2357-s002.pdf]
