# Supplementary material for: Multifunctionality is affected by interactions between green roof plant species, substrate depth, and substrate type
Source: Ecol Evol. 2017 Mar 11;7(7):2357–69. doi: 10.1002/ece3.2691 (PMC5383477; doi:10.1002/ece3.2691)

Figure S3. Average C/N ratio as a function of substrate depth and substrate type (±SE). Ratios were pooled for each family. Lower case letters indicate differences (p<0.05) between treatments within each family. Capital letters indicate differences (p<0.05) between families within each type/depth treatment.

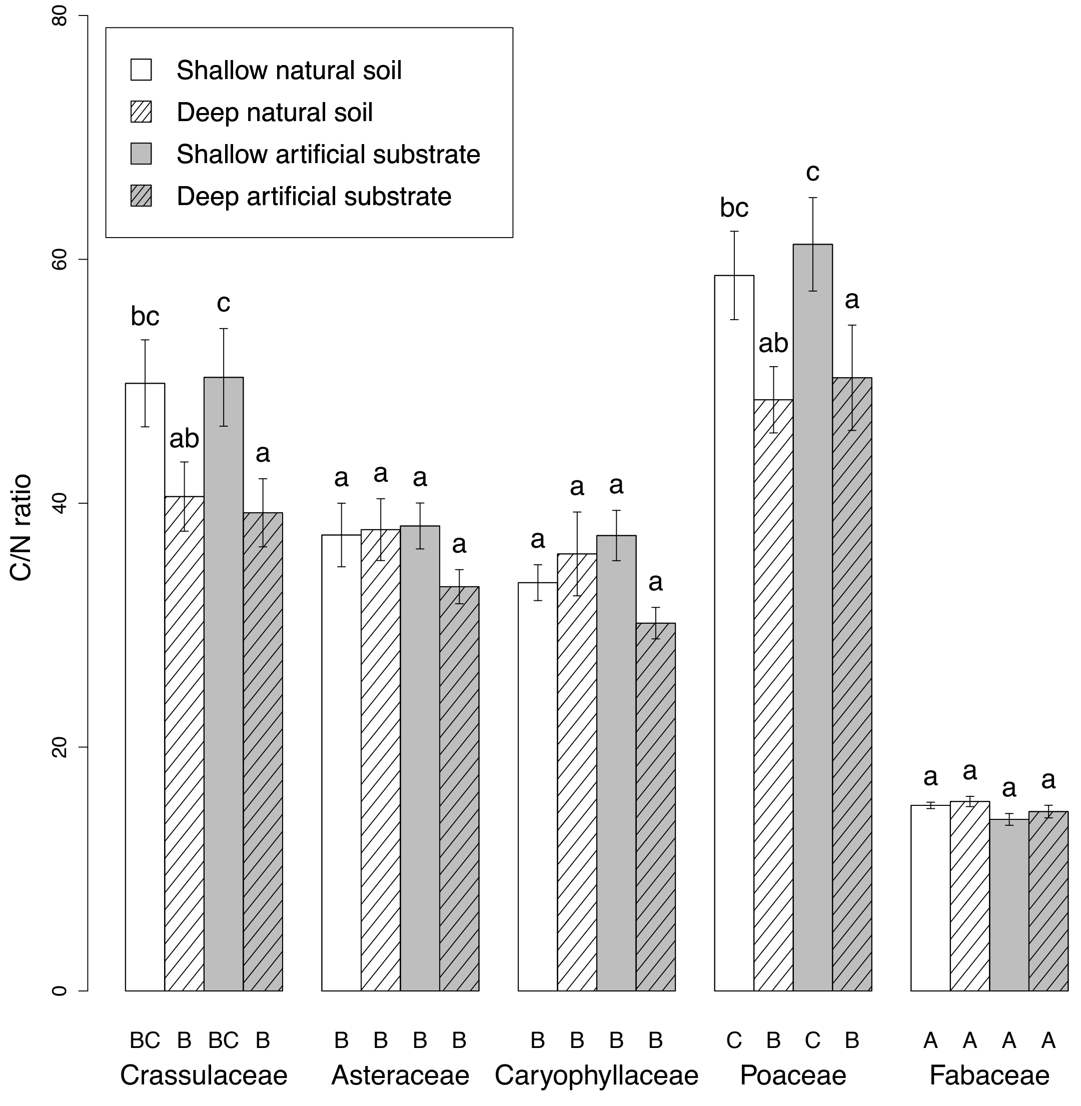

Supplement: Supplementary file 3 [file ECE3-7-2357-s003.pdf]
