# Supplementary material for: Multifunctionality is affected by interactions between green roof plant species, substrate depth, and substrate type
Source: Ecol Evol. 2017 Mar 11;7(7):2357–69. doi: 10.1002/ece3.2691 (PMC5383477; doi:10.1002/ece3.2691)

Figure S4. Retention curve for the substrates used in the experiment (±SE). Empty squares and dashed lines represent the natural soil. Full squares and full lines represent the artificial substrate. "Sat" stands for "saturation".

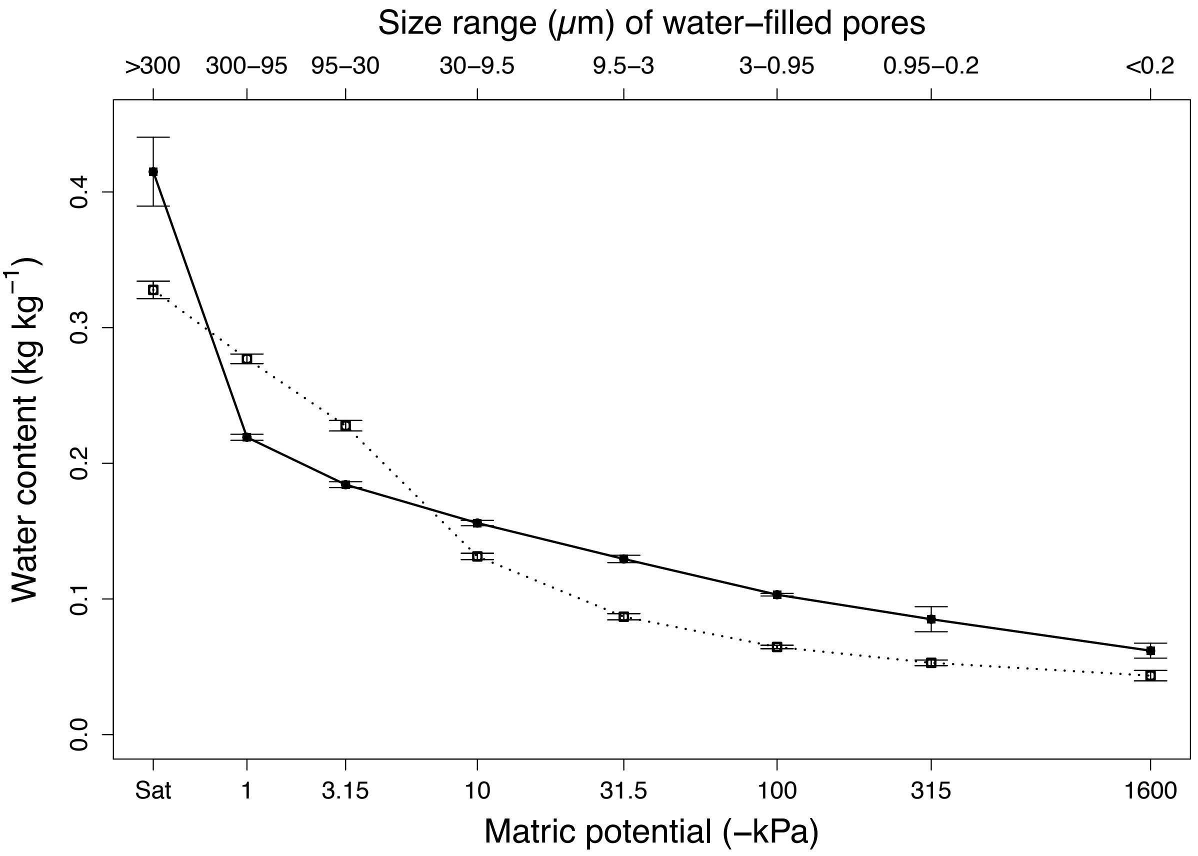

Supplement: Supplementary file 4 [file ECE3-7-2357-s004.pdf]
